# Origin–Destination Flow Estimation from Link Count Data Only

**DOI:** 10.3390/s20185226

**Published:** 2020-09-13

**Authors:** Subhrasankha Dey, Stephan Winter, Martin Tomko

**Affiliations:** Department of Infrastructure Engineering, University of Melbourne, 3010 Parkville, Victoria, Australia; winter@unimelb.edu.au (S.W.); tomkom@unimelb.edu.au (M.T.)

**Keywords:** urban road network, OD flow prediction, traffic count data, travel time estimation, microscopic simulation

## Abstract

All established models in transportation engineering that estimate the numbers of trips between origins and destinations from vehicle counts use some form of a priori knowledge of the traffic. This paper, in contrast, presents a new origin–destination flow estimation model that uses only vehicle counts observed by traffic count sensors; it requires neither historical origin–destination trip data for the estimation nor any assumed distribution of flow. This approach utilises a method of statistical origin–destination flow estimation in computer networks, and transfers the principles to the domain of road traffic by applying transport-geographic constraints in order to keep traffic embedded in physical space. Being purely stochastic, our model overcomes the conceptual weaknesses of the existing models, and additionally estimates travel times of individual vehicles. The model has been implemented in a real-world road network in the city of Melbourne, Australia. The model was validated with simulated data and real-world observations from two different data sources. The validation results show that all the origin–destination flows were estimated with a good accuracy score using link count data only. Additionally, the estimated travel times by the model were close approximations to the observed travel times in the real world.

## 1. Introduction

Origin–destination (OD) flow models estimate the number of vehicles in a given transportation network that are travelling between origins and destinations within a specific time interval [1]. Such estimations answer questions related to traffic congestion, and evaluate performances of theoretical models [2] and accessibility measures of public transport in urban areas [3]. OD flows can be estimated by traffic simulation from the travellers’ daily or weekly activity programs [4], or surveyed. Surveying OD flow data, however, is expensive in terms of time and effort, not scalable, and as far as household travel surveys are concerned, not comprehensive for all kinds of transport. Alternatively, OD flow can be estimated by statistical methods using two types of data: (i) using vehicle count data (link count data), and (ii) using either a sample of real-world OD flow data (e.g., historical trip data) using a non-Bayesian approach [5,6,7,8,9], or a prior belief about the OD flow (e.g., prior belief about the probability distribution of the OD matrix [10] or prior belief about inter-arrival rates [11]) in a Bayesian approach [11,12].

Historical trip data (or trajectory data) are collected from a variety of data sources, including GPS, Bluetooth, WiFi, GSM, and automatic number plate recognition (ANPR) sensors [13]. Trajectory data is highly privacy sensitive, hence, not always accessible, sometimes erroneous depending on the sampling rate, and incomplete and ambiguous with regard to the road network [14,15]. Some of the trajectory data, such as ANPR and Bluetooth data, are limited to static sensor locations [15]. These challenges around the trajectory data raise the question of whether a statistical OD flow model can be developed that does not need any trajectory data. Link count data, on the other hand, is collected from static sensors (e.g., inductive loop detectors, magnetic sensors), which are installed at either a road intersection or anywhere along a link of a road network [15,16]. Link count data are not privacy sensitive because only the number of vehicles in a road link is captured, without any other recorded identification. Link count data are less expensive to obtain and publicly available in many cities [17,18].

The estimated OD flow for non-Bayesian approaches is prone to be erroneous because the estimation depends on a subset of all the historical trips (observed or unobserved) completed in reality [5,7,8,19,20,21,22]. On the other hand, Bayesian statistical approaches in the literature rely on some prior beliefs about the probability distribution function of the OD flow and combine that belief with link count data to estimate OD flows [10,12]. Estimation using the Bayesian beliefs sometimes depends on prior knowledge of the sample of trip data to increase accuracy of estimation [10,11,12,23]. However, the quality of OD flow estimation remains uncertain for such OD flow estimations due to the lack of complete validation datasets. Existing literature validates an OD flow model compared either with a sample of known OD flow obtained from surveys or other sources of historical trip data [1,19,24], or with simulating synthetic data [10,12,23]. Surveying a complete OD flow is expensive, and is not feasible for the real world on a city scale. On the other hand, validating OD flow models using historical trip data cover only samples. Synthetic data are not guaranteed to include the complex nature of real-world traffic. Hence, there exists no sufficient validation in the literature.

In this paper, we provide a method for OD flow estimation that overcomes this limitation of existing methods: it no longer requires historical and either privacy-sensitive or commercially-sensitive—hence hard to get access to—trip data to provide OD flow and historical travel times for individual vehicles. Our method estimates OD flows only from link count data observed at road intersections. Our model can also estimate the travel time of individual vehicles in the road network from the estimated OD flows. Additionally, we propose a new validation technique for the estimated OD flow that compares the estimated travel time with real-world travel time data, which is available from reliable open data sources (e.g., [25]). We have used the network tomography model, a statistical OD flow model. The model was originally proposed as an Internet network tomography model [26] to estimate Internet traffic (OD flow) from packet counts observed at the network nodes. The network tomography model has since then been used in transportation networks already [11,12,23]. However, the prior transfers of the model to transportation networks had to make some assumptions about the real world [11,12,23]. Further, these methods do not consider the constraints of physical movement of the vehicles on the road and assume non-congested networks, and thus, ignore the principles of time geography [27]. Hence we propose a new transport network tomography model that estimates OD flows of the vehicles among all possible OD pairs only from link count data, and thus minimises prior assumptions. We provide theoretical proofs wherever necessary. We further implemented this method on a part of the inner-city road network of Melbourne, Australia. The model can be used elsewhere, requiring only an OpenStreetMap (OSM) database and link count data. To summarise, this paper makes the following contributions:

1. We estimated OD flows in a road network using link count data only, thereby overcoming the requirement for historical trip data and dependency on prior beliefs of any unobserved events. We also provide the necessary constraints and conditions of time geography for existing OD flow models for transportation network.
2. We integrated a microscopic simulator to incorporate physical movement of traffic into a road-network. Further, this enables us to evaluate travel times for individual vehicles in the estimated OD flow.
3. The traditional validation techniques are necessary but not sufficient, as there are multiple solutions [10]. We provide an additional validation technique for the estimated OD flows. We compared the estimated travel times with observed travel times from real-world data to evaluate the quality of our estimated OD flow.

## 2. Literature Review

A detailed review on statistical estimations of OD matrices suggests four main approaches for static systems [20]:

1. Minimum information/maximum entropy [5].
2. Maximum likelihood (ML) estimation of OD by sampling survey data and link counts [28].
3. Bayesian inference (BI) methods using a Bayesian estimator for the OD matrix, wherein the prior distribution of the trip matrix and the observed link count are assumed to have multivariate normal distributions [10,11,12].
4. Generalised least squares (GLS) estimation of the OD matrix [29] and enhanced estimation using constrained generalised least-squares [19].

Non-Bayesian approaches (e.g., Van Zuylen and Willumsen [5]) require a prior estimation for the OD matrix to be able to estimate OD flow from link count data. As an example, the derived methodology by [5] depends on $t_{ij}$ which is the a priori number of trips between *i* and *j* provided, where *i* and *j* are the origin and destination. The estimated OD flow is prone to be erroneous because the estimation depends on a historical trip dataset that contains a subset of all the trips (observed and unobserved) completed in reality in non-Bayesian approaches [7,8,21]. Constrained regression has also been proposed to estimate OD matrices [30]. Among the many statistical approaches, consideration of the stochastic nature of traffic counts has been also proposed to find out the link choice proportions in the estimation of the OD matrix [23]. All of these statistical methods require prior OD estimates and sets of sampled trajectory data [6]. The prior OD estimates may be found from either survey data or mathematical modelling, or a combination of both [31]. Real-time estimations and predictions of time-dependent OD flows have been developed and evaluated with real traffic data using state space models, but the estimation is dependent on the assignment matrix [32]. OD flows can also be estimated without a prior OD matrix under the assumption of a quasi-dynamic framework [1,7,9]. However, quasi-dynamic estimation techniques also require a specific setting of the transportation network where path choice is not an issue (e.g., highways or urban areas) [1]. In a quasi-dynamic traffic network, a satisfactory estimation of the parameters cannot be obtained when the number of OD pairs is significantly larger than the number of links in the transportation network [22]. Additionally, such a quasi-dynamic framework assumes that the path choices are identical with the trips generated from a certain origin of a traffic network at a certain time of the day on days of a particular category [1]. Hence, these models heavily rely on prior knowledge of historical trip data, for their traffic flow estimation from daily link counts. Another approach to estimating OD flows by characterising origin-flows (i.e., the number of vehicles originating from different origins in the network) leads to dimensionality reduction from $O(n^{2})$ to O(n) [14]. This approach has not been implemented and validated with real transportation data. Additionally, evaluating travel time from OD flows has not been discussed in the approach.

A comprehensive analysis on the impacts of different kinds of information to the OD flows estimation has shown the importance of a sample of path travel time along with the measure of flows on link sections [33]. Hence, a significant amount of work has been done on estimating OD flows and travel time predictions using data-based approaches. Some OD flow models are also capable of predicting travel time along with OD estimation. As an example, Bluetooth-based traffic monitoring for forecasting travel time and estimation of dynamic OD has been developed for freeways [34]. Additionally, a knowledge-based real-time travel time prediction system for urban networks has been developed that uses the raw data from a location-aware information system [35]. However, these data are highly privacy sensitive and sometimes erroneous [14]. Additionally, the unobservability of OD flow makes it difficult to evaluate the quality of the prediction, because the number of unknowns (i.e., OD flows) is much larger than the number of equations in the network (e.g., traffic counts) [7,22,36]. Previous literature has suggested using speeds and/or travel times (along with link counts) as traffic flow measurement input for dynamic OD flow estimation [8,32]. These estimation approaches use data sources including Bluetooth, ANPR, and mobile phone tracking [13], and we have discussed the issues of using such data sources for OD flow estimation. The stochastic nature of traffic counts, collected from multiple days, was considered first by Vardi [26] in order to estimate OD flows from link count data. Vardi [26] derived the full likelihood function from the link count data using a non-Bayesian approach. Vardi [26] introduced network tomography to derive the correct form of the full likelihood while considering inter-link dependency in the network and provided an independent Poisson assumptions-based origin–destination flow estimation technique [26]. However, Vardi [26] developed the network tomography model to estimate Internet traffic from packet counts observed at the graph nodes, which means Vardi’s modelling of route choice behaviour was principally designed for computer networks, and thus did not provide any route choice procedures that would be appropriate for modelling road traffic systems.

Vardi’s [26] network tomography has been extended in transportation research to demonstrate the feasibility of OD flow estimation only from link count data in a non-congested network [11,23] and independent Poisson assumptions [12]. Lo et al. [23] calculated the likelihood assuming that link flows are independent Poisson random variables, ignoring inter-link dependency [23]. Error in link choice proportions is imparted in the inferential process of OD flow estimation using a route choice probability matrix [23]. Hence, Hazelton [11] rejected the idea of estimating a route choice probability matrix exclusively from link data [11]. He estimated the route choice probabilities ($P_{hazelton}$) and OD flows ($\lambda_{hazelton}$) using log-likelihood functions of the joint distribution of the link count data ($X_{hazelton}$) with the assumption of an appropriate multivariate normal (MVN) distribution. He also discussed the limitations of his assumptions in practice for evaluating this log-likelihood function. Additionally, he suggested using a rough approximation of a log-likelihood function using a generalised least squares approach to estimate the route choice probability matrix and compare that with the MVN distribution. Further, Hazelton [11] proposed a new method to approximate the probability distribution function of measurement error $f_{Z}\left(z|\lambda ,P\right)$ as an MVN distribution $N(0,\Sigma_{Z})$ for estimating measurement error *Z* as parameter of inter-arrival rate (λ), route choice matrix (*P*), and other parameters ψ. Thus, the extension of the network tomography approach includes many assumptions and uncertainties in the estimation process due to unknown values of *P* and λ in the current context of estimating OD flow only from link count data. Vardi [26] pointed out that the MVN approximation and central limit theorem’s applicability depends on the value of *K*, i.e., the number of observation periods, and thus the MLE estimation of the joint distribution $f_{Z}\left(z|\lambda ,P\right)$ is a poor approximation. Hazelton [24] implemented his model in a regional area of Leicester with error-prone traffic count data. However, there remain two issues in the implementation: (i) the use of prior information of survey data to find the route choice probabilities, and (ii) the joint probability distribution of the link counts and variation in route choice probabilities of the vehicles are derived under independent Poisson distributed OD flows. Tebaldi and West [12] have pointed out that independent Poisson assumptions are questionable in many contexts of traffic flow estimation problems. Tebaldi and West [12] contributed to point out theoretical deficiencies in likelihood and other non-Bayesian approaches that add biases in the inferred OD flows due to structural determinants in transportation networks [12]. The Bayesian approaches assume some prior beliefs in estimating the OD flows [10,11,12]. The existing network tomography has limitations while applied in transportation networks. According to our previous discussions, there are three questions that remain unanswered when applying the network tomography model in the context of a transport network:

- There are limitations in other MLE estimation methods, as discussed by Vardi [26], Hazelton [11], and Tebaldi and West [12]. Hence, ref. [26] proposed to derive the first and second-order moments of *Y* to solve Equation (10). However, derivation of the first and second-order moments of *Y* is not theoretically proven for the Markovian routing.
- Vardi’s [26] network tomography assumes that the values of route choice probabilities are known in a computer network, and does not provide any derivation for a transport network.
- Poisson assumptions might be true at the origins, but are definitely not true at the destinations with the same parameter values, due to road traffic. Hence, estimated solutions, i.e., estimated inter-arrival rates between all OD pairs (Λ), need to be modified at the destinations.

In this paper our contributions are focused on overcoming these limitations with an additional validation from real-world observations that is inherently missing in the existing literature. We propose a new transport network tomography model that discards the independent Poisson assumptions using space time constraints at the destinations. However, we keep the Poisson assumptions of the constant traffic generation rates at the origin of an OD pair before being influenced by traffic using an exponential distribution modelling. We incorporate the effect of traffic congestion further using a microscopic simulator, and overcome the limitations of prior models estimating OD flow in non-congested networks. We also provide a probabilistic route choice modelling approach based on travel time [20] that considers behavioural aspects of drivers in a road network. Thus, we achieve estimating OD flows using only link count data as our first major contribution. The generalisation of the assessment of an OD estimation is a difficult task, with a large number of different methods described by researchers [6,7,8]. The sufficient condition of the OD flow estimation is possible only when one has the prior information about every possible OD flow on the road network (which is a next to impossible proposition). Hence we propose to use a validation technique that overcomes these limitations using real-world observations, a validation that is inherently missing in previous articles. Movement in a city may be validated using travel times from historical trip data [33,37]). Hence, OD flow model estimated travel times can be compared using observed travel time data in the real world [12]. However, no methodology has been proposed previously to validate any OD flow model using travel times. Hence, as our second major contribution, we estimate travel times of individual vehicle from the estimated OD flow, and we validated these estimated travel times with real-world observations.

## 3. Theory

### 3.1. Network Tomography

Let *G* be a directed weighted road network graph whose nodes represent the road intersections and whose edges represent the road segments (links). If there are *n* nodes in the graph, then there will be J=n(n−1) possible OD pairs. OD pair *j* indicates a unique OD pair among the set of *J* possible OD pairs. We define a parameter, the inter-arrival rate λ, that indicates the number of vehicles travelling from an origin to a destination within an observation period of *T* minutes. If the arrival rate of cars between a fixed origin and destination is constant for *T* minutes, then the arrival of the vehicles within the small interval can be approximated using a Poisson process. Let *K* be the total number of observed days and *I* be the number of links in the network graph. Let ${X_{j}}^{k}$ be the number of cars travelling between an OD pair *j* for a fixed observation period of *T* minutes on $k^{\mathrm{th}}$ day.

(1) $${X_{j}}^{k}∼Poisson\left(\lambda_{j}\right)\;\mathrm{where}\;j=1,2,\ldots ,J\;\mathrm{and}\;k=1,2,\ldots ,K$$

Let Λ be a vector that contains the inter-arrival rate of vehicles for all *J* ODs, with $\lambda_{j}$ the inter-arrival rate of the $j^{\mathrm{th}}$ OD:

(2) $$\Lambda ={\left(\lambda_{1},\cdots ,\lambda_{j},\cdots ,\lambda_{J}\right)}^{⊤}$$

Let $Y^{k}$ represent the individual link count data vector and $X^{k}$ the trip vector for a particular OD pair in the network at the $k^{\mathrm{th}}$ period respectively. Then:

(3) $$X^{k}={\left({X_{1}}^{k},\cdots ,{X_{j}}^{k},\cdots ,{X_{J}}^{k}\right)}^{⊤}$$

(4) $$Y^{k}={\left({Y_{1}}^{k},\cdots ,{Y_{i}}^{k},\cdots ,{Y_{I}}^{k}\right)}^{⊤}$$

Vardi [26] assumed that the network has an initial path-link incidence matrix A (I×J) such that:

(5) $$\begin{array}{c} Y^{k}=AX^{k}\;\mathrm{where}\;k=1,2,\ldots ,K\\ a_{i}^{j}\left\{\begin{array}{cc} =1 & \mathrm{if}\;\mathrm{link}\;i\;\mathrm{is}\;\mathrm{only}\;\mathrm{link}\;\mathrm{of}\;\mathrm{OD}\;\mathrm{pair}\;j\\ =0 & \mathrm{otherwise} \end{array}\right. \end{array}$$

In Equation (5), $a_{i}^{j}$ represents the element in the i,j position of a fixed routing matrix A. However, Equation (5) is highly under-constrained and has multiple solutions (as I<J for n>2). One of the solutions is the maximum likelihood estimation (MLE) of the whole system of linear equations. Hence, MLE is the popular choice of estimating *X* from *Y* observing for *K* number of days with the Poisson assumptions within the time period *T*. As an example, the Poisson assumption is expected to be true from 9:00 AM to 9:15 AM of every weekday of the first quarter of an observational period. Vardi [26] has provided the derivative of the log-likelihood $\partial \log(\mathrm{Likelihood})/\partial \lambda_{j}$ for j=1,⋯,J in vector notation:

(6) $$\frac{\partial l}{\partial \lambda_{j}}=\frac{1}{K}\sum_{k=1}^{K}E_{\lambda }\left[X^{k}|Y^{k}={\mathrm{AX}}^{k}\right]-\Lambda$$

The MLE estimate from Equation (6), $\Lambda_{MLE}$, is sought numerically using expectation-maximisation (EM) [38] to maximise *l*. Vardi [26] pointed out the computational limitations in solving Equation (6), which may converge to a non-MLE estimate. He has also shown that likelihood equations might have a unique solution. However, that solution can differ from the MLE solution that also may be unique.

Apart from the fixed routing matrix A, ref. [26] considered $a_{i}^{j}$ as link choice probabilities. Link choice probability $a_{i}^{j}$ is defined as the proportion of incoming traffic likely to travel through link *i* for an OD pair *j* (here, *j* is a number that indicates a unique OD pair) without the knowledge of the path taken to arrive at link *i* (memory-less Markovian assumptions). Thus, Equation (5) becomes:

(7) $$\begin{array}{c} Y^{k}=AX^{k}\;\mathrm{where}\;k=1,2,\ldots ,K\\ a_{i}^{j}\left\{\begin{array}{cc} =1 & \mathrm{link}\;i\;\mathrm{belongs}\;\mathrm{to}\;\mathrm{the}\;\mathrm{only}\;\mathrm{path}\;\left(\mathrm{route}\right)\;\mathrm{of}\;\mathrm{the}\;\mathrm{OD}\;\mathrm{pair}\;j\\ <1 & \mathrm{link}\;i\;\mathrm{belongs}\;\mathrm{to}\;a\;\mathrm{possible}\;\mathrm{route}\;\mathrm{out}\;\mathrm{of}\;\mathrm{the}\;\mathrm{OD}\;\mathrm{pair}\;j\\ =0 & \mathrm{link}\;i\;\mathrm{does}\;\mathrm{not}\;\mathrm{belong}\;\mathrm{to}\;\mathrm{any}\;\mathrm{route}\;\mathrm{of}\;\mathrm{the}\;\mathrm{OD}\;\mathrm{pair}\;j \end{array}\right. \end{array}$$

In the real world, the routing matrix is neither fixed nor memory-less. Hence, the proportion of traffic, passing through a particular link *i* is different for different routes among all OD pairs. That means a link *i* might be more likely for a route with OD *j*, but less likely for another route of an OD $j^{\prime }$. We are interested in estimating these route choice probabilities $\left\{P_{j}^{i},P_{j^{\prime }}^{i},\cdots \right\}$ for every possible routes of each OD pair. Estimating these $P_{j}^{i}$ will result in a Markovian routing matrix **P** with finite memory such that Equation (7) becomes:

(8) $$Y^{k}=PX^{k}$$

In Equation (8), $P_{i}^{j}$ is the route choice probability of *i*, considering all possible routes that involve link *i* for the OD pair *j*. Thus, $P_{i}^{j}$ are the route choice probabilities with the knowledge of the path taken to arrive at link *i* (Markovian assumptions with finite memory). The finite-memory Markovian routing matrix P of the network can be obtained from the initial routing matrix A. Considering all the links with positive probability for a specific OD pair as active links (M), a Markov chain with finite states can be built for each OD pair *j*. $Q^{\left(m\right)}$ is then the $m^{\mathrm{th}}$ order state transition matrix of the chain. $Q^{\left(m\right)}$ is a (M+1)×(M+1) matrix. Let the initial probability of the chain for an OD pair be $\pi^{j}$, then ${\pi_{i}}^{j}={P_{i}}^{j}$ and $\pi_{end}=0$.

(9) $${P_{i}}^{j}={\pi_{i}}^{j}\sum_{state=0}^{M}{\left[Q^{\left(j\right)}\right]}^{state}$$

As discussed in Section 2, Hazelton [11] extended the network tomography for transportation networks using a Bayesian approach. He estimated the route choice probabilities ($P_{hazelton}$) and OD flows ($\lambda_{hazelton}$) using log-likelihood functions of the joint distribution of the link count data ($X_{hazelton}$) with the assumption of an appropriate multivariate normal distribution. He also discussed the limitations of these assumptions in practice for evaluating this log-likelihood function. Limitations of such an approach were also pointed out earlier by [26] in the context of solving Equation (6). Hence, ref. [26] estimated the solution of Equation (8) using the first and second-order moment method. He inferred that mean and variance of *Y* will be equal to the dot product of the Markovian routing matrix and estimates of inter-arrival rates:

(10) E[Y]=PΛandVar[Y]=PΛ

However, Equation (10) is still highly under-constrained. Hence, ref. [26] suggested, additionally, the use of higher order statistics of the link count data to create additional constraints for solving this equation as a better approach to estimate OD flows compared to MLE of the Poisson parameters. Let $i^{\prime }$ have the same domain of *i*. As $Y_{i}$ and $Y_{i^{\prime }}$ are independent Poisson random variables (with *K* observations), Vardi [26] proved that for fixed routing matrix A:

(11) $$\begin{array}{c} S_{ii^{\prime }}=Cov\left(Y_{i},Y_{i^{\prime }}\right)=\sum_{j=1}^{J}B_{ii^{\prime },j}X_{j}\\ \;\mathrm{where}\;B_{ii^{\prime }}^{j}=a_{i}^{j}\cdot a_{i^{\prime }}^{j}\;\mathrm{and}\;E\left[Y_{i}\right],S_{ii^{\prime }}\geq 0 \end{array}$$

where $S_{ii^{\prime }}$ is the ${\left(ii^{\prime }\right)}^{\mathrm{th}}$ element of the column vector S, and $B_{ii^{\prime }}^{j}$ is the ${\left(ii^{\prime },j\right)}^{\mathrm{th}}$ element of a matrix **B** that can be obtained from matrix A. Thus, Equation (5) will be supplemented with S=BX. Hence, the final equation for the estimation will be the following:

(12) $$\left[\begin{array}{c} E[Y]\\ S \end{array}\right]=\left[\begin{array}{c} A\\ B \end{array}\right]\Lambda$$

If *R* is the number of rows in the co-variance matrix B, then $\lambda_{j}$ will be updated after each iteration according to the following formula of the EM algorithm [26]:

(13) $$\lambda_{j}^{n+1}\leftarrow \frac{\lambda_{j}^{n}}{\sum_{i=1}^{I}p_{ij}+\sum_{i=I+1}^{I+R}b_{ij}}\left(\sum_{i=1}^{I}\frac{p_{ij}E\left[Y_{i}\right]}{\sum_{l=1}^{J}p_{il}\lambda_{l}^{n}}+\sum_{i=I+1}^{I+R}\frac{b_{ij}.S_{i}}{\sum_{l=1}^{J}b_{il}\lambda_{l}^{n}}\right)$$

where $\lambda^{n+1}$ is the ${\left(n+1\right)}^{\mathrm{th}}$ iteration and depends on the value $\lambda^{n}$ obtained at $n^{\mathrm{th}}$ iteration.

### 3.2. Transport Network Tomography

We have already discussed in Section 2 that there are three limitations of the existing network tomography model in transport networks:

- Derivation of the first and second-order moments of *Y* is not theoretically proven for the Markovian routing.
- Derivation of the Markovian routing matrix P is not provided for a transport network.
- Independent Poisson assumptions need to be modified at the destinations due to space-time constraints.

We will now discuss our proposed model, the transport network tomography, which overcomes the above mentioned limitations.

#### 3.2.1. Estimation Using First and Second-Order Moments

We provide a methodology to derive the first and second-order moments of Y in order to estimate the solutions of Equation (8) without the independent Poisson assumptions of {X}. $Y_{i}$ is a random variable (RV) of the link count observed at link *i*. From Equation (8), $Y_{i}$ is a linear combination of multiple RVs $\left\{X_{1},\cdots ,X_{J}\right\}$ and route choice probabilities, $P_{i}^{j}$s. Each link count RV $Y_{i}$ depends on the product of route choice probability $P_{i}^{j}$ and the probability of $X=X_{j}$, where $Pr\left(X=X_{j}\right)=\left\{\frac{\lambda_{j}}{\sum_{j=1}^{J}\lambda_{j}}\right\}$. Thus:

(14) $$\begin{array}{cc} Y_{i} & =P_{i}^{1}X_{1}+\cdots +P_{i}^{j}X_{j}+\cdots +P_{i}^{J}X_{J}\\ & =\sum_{j=1}^{J}P_{i}^{j}X_{j} \end{array}$$

With $\left\{P_{i}^{1}X_{1},\cdots ,P_{i}^{j}X_{j},\cdots ,P_{i}^{J}X_{J}\right\}$ as random variables with probabilities $\frac{P_{i}^{1}\lambda_{1}}{\sum_{j=1}^{J}P_{i}^{j}\lambda_{j}},\cdots ,\frac{P_{i}^{j}\lambda_{j}}{\sum_{j=1}^{J}P_{i}^{j}\lambda_{j}},\cdots ,$ $\frac{P_{i}^{J}\lambda_{J}}{\sum_{j=1}^{J}P_{i}^{j}\lambda_{j}}$. Hence, RV $\left\{Y_{i}=\sum_{j=1}^{J}P_{i}^{j}X_{j}\right\}$ follows a multinomial distribution:

(15) $$\left\{Y_{i}=\sum_{j=1}^{J}P_{i}^{j}X_{j}\right\}∼\mathrm{Multinomial}\left(\sum_{j=1}^{J}P_{i}^{j}X_{j},p_{i}\right)$$

(16) $$p_{i}=\left(\frac{1}{\sum_{j=1}^{J}P_{i}^{j}\lambda_{j}}\right)\mathrm{diag}\left(\Lambda^{T}\right)\cdot P_{i}$$

where $p_{i}$ is a column vector with a dimension of (J,1) for j=1,⋯,J, such that the $j^{\mathrm{th}}$ element of $p_{i}$ is $p_{i}^{j}=\left\{\frac{P_{i}^{j}\lambda_{j}}{\sum_{j=1}^{J}P_{i}^{j}\lambda_{j}}\right\}$, and $P_{i}$ is a row vector $\left\{P_{i}^{1},\cdots ,P_{i}^{J}\right\}$ with dimension of (1,J). Now, we can write the expectation of RV $Y_{i}$:

(17) $$\begin{array}{cc} E\left[Y_{i}=P_{i}X\right] & =\sum_{j=1}^{J}\left\{Y=\sum P_{i}^{j}X_{j}\right\}\cdot Pr\left(X=P_{i}^{j}X_{j}\right)\\ \mathrm{Or},\;E[Y_{i};\Lambda ] & =\sum_{j=1}^{J}\left(\sum_{j=1}^{J}P_{i}^{j}\lambda_{j}\right)p_{i}^{j}\\ & \sum_{j=1}^{J}\left(\sum_{j=1}^{J}P_{i}^{j}\lambda_{j}\frac{P_{i}^{j}\lambda_{j}}{\sum_{j=1}^{J}P_{i}^{j}\lambda_{j}}\right)\\ & =\sum_{j=1}^{J}P_{i}^{j}\lambda_{j}\\ & =P_{i}\cdot \Lambda \end{array}$$

where $P_{i}$ is the $i^{\mathrm{th}}$ row of P corresponding to $Y_{i}$. Link count data are random vectors $Y={\left\{Y_{1},\cdots ,Y_{i},\cdots ,Y_{I}\right\}}^{T}$, and from the properties of random vectors we get:

(18) $$E\left[Y\right]=\left[\begin{array}{c} E[Y_{1}]\\ E[Y_{2}]\\ \vdots \\ E[Y_{I}] \end{array}\right]=\left[\begin{array}{c} P_{1}\\ P_{2}\\ \vdots \\ P_{I} \end{array}\right]\Lambda =P\Lambda$$

If Cov(Y) and Cov(X) are the co-variances of *Y* and *X* respectively for Y=PX, then using linearity of expectations:

(19) $$\begin{array}{cc} Cov(Y) & =\mathrm{PE}\left[\left(X-\mathrm{EX}\right){\left(X-\mathrm{EX}\right)}^{T}\right]P^{T}\\ \; & =PCov\left(X\right)P^{T} \end{array}$$

For two different links *i* and $i^{\prime }$, we can evaluate co-variance $S(i,i^{\prime })$ from Equation (19) as follows:

(20) $$\begin{array}{cc} S_{i,i^{\prime }} & =Cov(Y_{i},Y_{i^{\prime }})\\ & =P_{i}\cdot \mathrm{diag}\left(\Lambda^{T}\right)\cdot {P_{i^{\prime }}}^{T}\\ & =\sum_{j=1}^{J}P_{i}^{j}\cdot P_{i^{\prime }}^{j}\cdot \lambda_{j} \end{array}$$

Thus, Equation (20) is derived for a Markovian routing matrix *P*, which holds true for Equation (11) for a Fixed routing matrix A as derived by [26]. We calculate $\left(ii^{\prime },j\right)$ elements of matrix B (see Equation (11)) with the relation $B_{ii^{\prime }}^{j}=P_{i}^{j}\cdot P_{i^{\prime }}^{j}$. Thus, our final Equation for deriving OD flow with Markovian routing reduces to:

(21) $$\left[\begin{array}{c} E[Y]\\ S \end{array}\right]=\left[\begin{array}{c} P\\ B \end{array}\right]\Lambda$$

It should be noted that Equation (21) is similar to Equation (12), which was proposed by [26] with the assumption of independent Poisson assumptions for a fixed routing matrix A. We have provided a theoretical proof of Equation (21) without the independent Poission assumption for a Markovian routing matrix **P**. For an observation period of *K* days, $E\left[Y_{i}\right]=\frac{1}{K}\sum_{k=1}^{K}Y_{i}^{k}$ can be calculated from the link count data. We can also derive B,S using Equation (20). Once we derive the Markovian routing matrix P, we can use Equation (21) to derive the estimate Λ using Equation (13). Hence in the next subsection, we will discuss the methodology for deriving P.

#### 3.2.2. Derivation of the Markovian Routing Matrix

In Section 3.1 we have explained that the route choice probabilities of a link *l* for an OD pair *j* ($P_{j}^{i}$) depend on the conditional probabilities of the link choice probabilities $a_{i}^{j}$. Hence, an initial routing matrix A consists of the probability of a vehicle to travel only from the current node to all the adjacent nodes towards its destination (memory-less Markovian routing). In a grid-like urban road network, there exist multiple shortest paths (by minimum travel time) and other longer paths from an origin to a destination. Hence, we propose the following equation for calculating the initial probabilities of a vehicle traveling from a node Cur to node $Next^{r}$. If there are *R* outgoing links from node Cur, then 1<=r<=R:

(22) $$P_{Next^{r}}=C\times \exp(-2|\frac{t_{r}-t_{min}}{t_{min}}\left|\right)$$

where $t_{min}$ is the average time required to get from Cur to the destination, and $t_{r}$ is the time taken via $Next^{r}$. If $Next^{r}$ is an intermediate node then these probabilities will add up to 1:

(23) $$\sum_{r=1}^{R}P_{Next^{r}}=1$$

Thus, we get the value of *C* and initial probabilities by solving Equation (23) and further use it to create the initial routing matrix as shown in the following equation:

(24) $$A=\mathrm{Links}(I)\overset{\mathrm{OD}\;\mathrm{pairs}(J)}{\left[\begin{array}{cccc} a_{11} & a_{12} & \cdots & a_{1J}\\ a_{21} & a_{22} & \cdots & a_{2J}\\ \vdots & \vdots & \ddots & \vdots \\ a_{I1} & a_{I2} & \cdots & a_{IJ} \end{array}\right]}$$

where $a_{ij}$ are the initial probabilities calculated from Equations (22) and (23) for OD pair *j* that involves link *i*. Once we derive *A*, we can derive P using Equation (9). Finally, we can use Equation (21) to derive the estimate Λ using Equation (13).

#### 3.2.3. Space-Time Constraints

Once we estimate Λ, we constrain our model to the conditions of physical movement. We add the following assumptions and concepts from time geography:

1. In an urban road network, we assume that each road intersection can be an origin or destination for a vehicle that has been counted at least once by any traffic counting sensor installed in the same road network [31].
  (This assumption was adopted from Internet traffic, and a sufficient approximation that a vehicle is at least counted once to be observable at the link count data).
2. The traffic generation rate at the origins of the vehicles can be assumed to be constant over a small interval of time. This assumption stems from a continuous traffic flow function over the course of a day that can be approximated as constant over small periods of time.
  (In contrast to the network tomography, this assumption only assumes a Poisson distribution at the origins and not at the destination).
3. Within this time interval, a vehicle must stay inside the space-time cone [39] formed by its origin and physical constraints of space and speed of travel, guaranteeing that its current location is reachable within the time budget.
  (Physical travel constraints do not apply for Internet traffic).
4. All vehicles may travel along any of the possible routes available from origin to destinations with different probabilities. These probabilities are calculated based on minimum travel time required to travel between two adjacent nodes. In our model, we have assumed higher travel time leads to lower probability of travel. We have calculated the minimum travel time of an origin to a destination using the distances between the OD pair and speed-limit in that shortest route of the graph for that OD pair. The initial routing matrix is built first based on minimum travel time between two adjacent nodes. Then the Markovian routing matrix has been calculated from the initial routing matrix.
  (Distance and travel time do not matter in Internet traffic).
5. The vehicles’ travel distances can be safely assumed to be larger than the distances between neighbouring urban road intersections, i.e., at least one full link long.
  (Internet traffic has no minimal distances between OD for existence).

Hence, we add the space-time constraints to model the estimated inter-arrival rates among OD pairs. The time-gap between arrival of each vehicle can be approximated using an exponential distribution. If $\delta_{T}$ is the time-gap between two vehicles with the same OD pair *j* and rate of traffic generation at the origin $\lambda_{j}^{origin}$, and $t_{average}$ is the average travel time for a vehicle to reach its destination from the origin, then:

(25) $$\delta_{T}=\left(T-t_{average}\right)/\lambda_{j}^{origin}\;\mathrm{where}\;t_{average}=\mathrm{Distance}/\mathrm{average}\mathrm{speed}$$

However, in transport network tomography the rate at which vehicles are arriving at destinations is not equal to the rate at which they are generated at the origins. Thus, in the real world, $\lambda_{j}^{origin}\neq \lambda_{j}^{destination}$ for an OD pair *j*. Hence we alternatively define our parameter of estimation, $\lambda_{j}$, as traffic generation rate at the origin of OD pair *j*, instead of inter-arrival rate of the same OD pair *j*. Thus, we get $\lambda_{j}^{origin}=\lambda_{j}\neq \lambda_{j}^{destination}$.

### 3.3. Transport Network Tomography: An Illustrative Example

**Figure 1:**
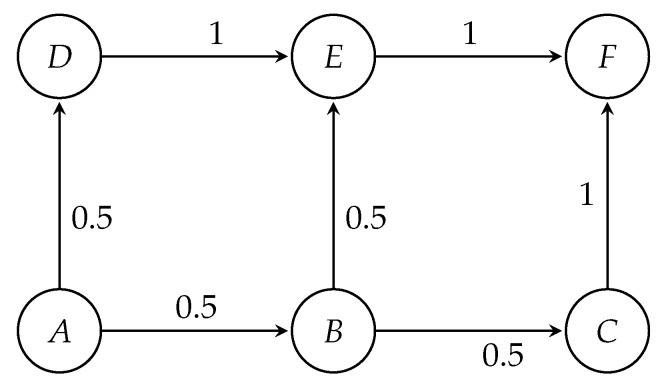
A toy network to illustrate the transport network tomographic model.

Let us now explain the transport network tomography model with a toy network as an illustrative example. Let there be six road intersections *A*–*F* in the network in Figure 1.

The initial routing probabilities shown in the figure were calculated based on Equation (22). For simplicity let us assume that the routes ABEF, ABCF, and ADEF have the same average travel time. If Cur is *A*, then *B* and *D* are adjacent, or the Next nodes of *A*. Hence, we consider only those shortest paths that include the adjacent nodes of *A*; i.e., we only consider links AB and AD. By putting these values in Equations (22) and (23), we obtain the following initial probabilities for node *A*:

(26) $$\begin{array}{c} P_{AB}=C\times \exp\left(-2\times 0\right)=C\\ P_{AD}=C\times \exp\left(-2\times 0\right)=C\\ P_{AB}+P_{AD}=1\\ or,\;2C=1\\ or,\;C=0.5 \end{array}$$

Thus:

(27) $$A=\begin{array}{cc} & \begin{array}{cccc} OD_{1} & OD_{AF} & \cdots & OD_{J} \end{array}\\ \begin{array}{c} AB\\ AD\\ BE\\ DE\\ BC\\ EF\\ CF \end{array} & \left(\begin{array}{cccc} \vdots & 0.5 & \cdots & \vdots \\ \vdots & 0.5 & \cdots & \vdots \\ \vdots & 0.5 & \cdots & \vdots \\ \vdots & 1 & \cdots & \vdots \\ \vdots & 0.5 & \cdots & \vdots \\ \vdots & 1 & \cdots & \vdots \\ \vdots & 1 & \cdots & \vdots \end{array}\right\} \end{array}$$

Next we calculate **P** from **A** using Equation (9).

(28) $$\begin{array}{cc} {P_{i}}^{j}= & \;\mathrm{the}\mathrm{probability}\mathrm{of}a\mathrm{vehicle}\mathrm{with}\mathrm{OD}\mathrm{pair}j\mathrm{passing}\mathrm{through}\mathrm{link}i \end{array}$$

(29) $$\begin{array}{cc} {P_{EF}}^{AF} & =\;0.5\times 1\times 1+0.5\times 0.5\times 1\\ & =\;0.75 \end{array}$$

Thus, we get:

(30) $$P=\begin{array}{cc} & \begin{array}{cccc} OD_{1} & OD_{AF} & \cdots & OD_{J} \end{array}\\ \begin{array}{c} AB\\ AD\\ BE\\ DE\\ BC\\ EF\\ CF \end{array} & \left(\begin{array}{cccc} \vdots & 0.5 & \cdots & \vdots \\ \vdots & 0.5 & \cdots & \vdots \\ \vdots & 0.25 & \cdots & \vdots \\ \vdots & 0.5 & \cdots & \vdots \\ \vdots & 0.25 & \cdots & \vdots \\ \vdots & 0.75 & \cdots & \vdots \\ \vdots & 0.25 & \cdots & \vdots \end{array}\right\} \end{array}$$

With the derived value of **P**, now we can calculate **S** and **B** as per Equation (21) of our model, and evaluate Λ from Equation (13).

**Figure 2:**
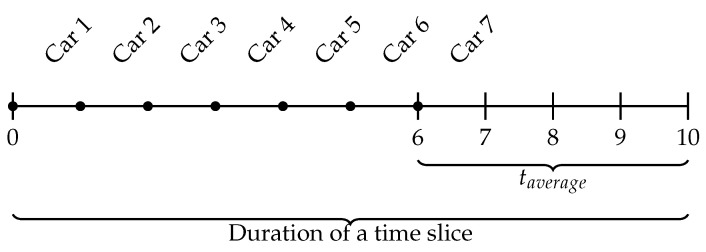
Space-time constraints: time gaps between two consecutive vehicles with the same origin and destination.

Let us now explain Equation (25) with a simple example. As illustrated in Figure 1, if the inter-arrival rate is seven cars within a 10 min interval between origin *A* and destination *F*, then $\lambda_{j}^{origin}=\lambda_{j}=7$. Let the average travel time $t_{average}$ from origin to destination be 4 min. Hence, then all vehicles must reach the destination before the time slice of 10 min ends. Figure 2 is illustrating how the traffic generation rate at the origin is converted to time-difference between two vehicles with the same origin and destination. Due to the exponential distribution, the rate of traffic originating only at the origin is constant for this observation period. Now within this time slice, every vehicle should be reaching its destination. Hence, we have to make sure that the last vehicle originating from the origin should reach the destination (or to be counted by the final sensor in that route) before the time slice ends. However, the rate at which the vehicles arrive at the destination is purely dependent on the physical conditions of movement and space-time constraints. This initialisation is created by a script and passed to a microscopic simulator that takes care of other transportation factors, such as traffic lights or speed limits, to compute actual travel times.

**Figure 3:**
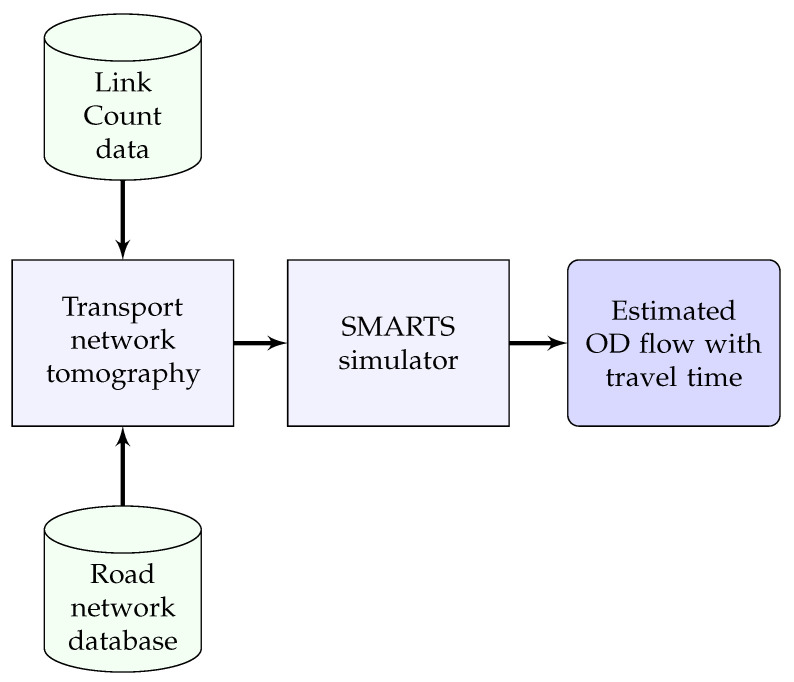
The block diagram of our transport network tomography model to estimate OD flows using link count data only.

Thus, transport network tomography produces rates of vehicles originating at the origins that are further processed in a microscopic traffic simulation platform, in our case SMARTS [40]. SMARTS accepts traffic data that contains trajectories to produce travel times of individual vehicles in the city traffic. Scheduled public transport vehicles are also integrated to increase the model’s accuracy because they provide constraints on the waiting time at intersections and delays in the network. Figure 3 shows the block diagram of the transport network tomography for estimating OD flows using link count data only.

## 4. Implementation

**Figure 4:**
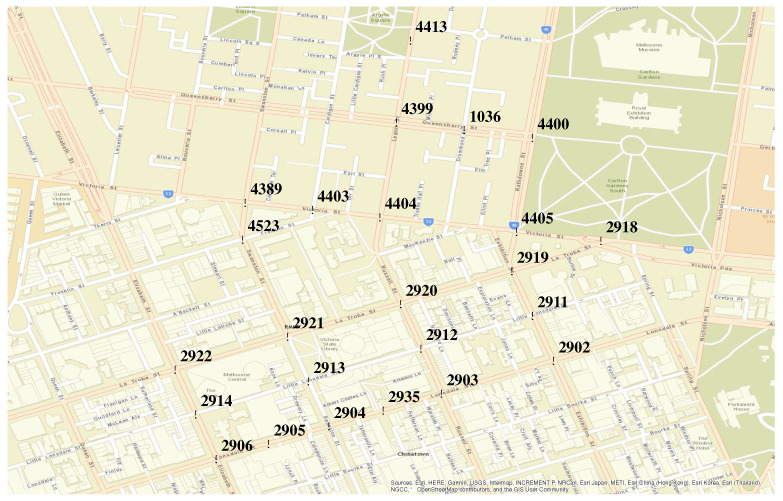
Area selected for the case study with traffic count locations.

The model has been implemented to estimate OD flows of the counted vehicles in the road intersections of a city road network. For reasons of availability of the link count data and validation data, the implemented traffic flow model has been tested over a small area of the central part of the City of Melbourne shown in Figure 4. This area is realistic, complex, and contains a mixture of different topological structures in terms of the road network. For this area, SCATS data are available through the Open Data initiative of the Victorian Government [17]. SCATS [17] is an urban traffic control system that maintains a database of traffic counts from loop detectors with the locations of the sensors. In the area shown, SCATS sensors are installed at each road intersection, and for each lane individually. The OSM database for Melbourne has been used to construct the detailed directed road network (OSM graph). The microscopic traffic simulation platform SMARTS [40] has been used to simulate the traffic flow using the model-generated time-stamped vehicle data and thus estimating travel time among all possible OD pairs.

### 4.1. Data Requirements

In the implemented model, both private and public transportation have been considered. The number of private transport vehicles are estimated using the network tomographic approach. Schedule, routes, and number of public transport vehicles were collected from the Public Transport Victoria (PTV) General Transit Feed Specification (GTFS) data [41]. The data required for implementation are the following:

1. OSM data for all the nodes and edges (with unique OSM IDs) of the road network.
2. Traffic count locations with unique SCATS IDs [41].
3. Traffic signal configuration data sheets [41].
4. Traffic signal volume data [41] collected at each road intersection as link count data.
5. PTV GTFS data [41].

### 4.2. Data Pre-Processing

**Figure 5:**
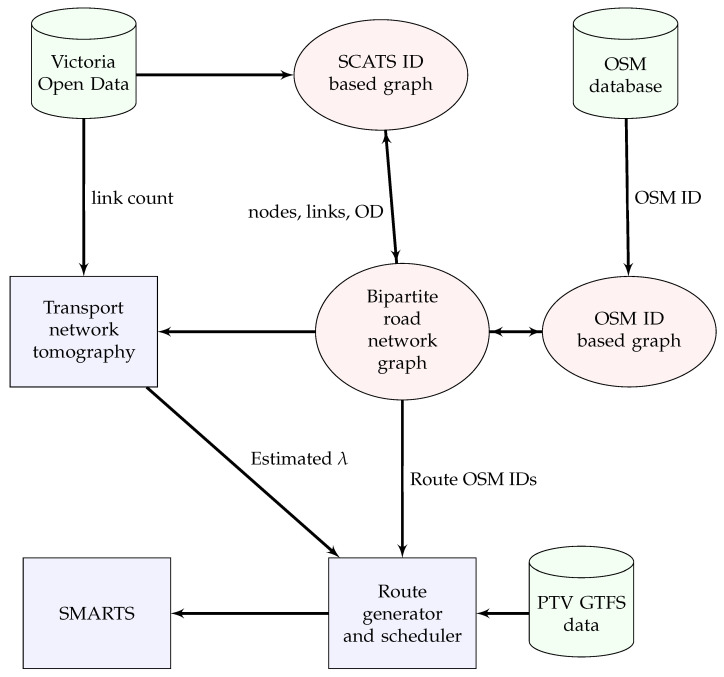
Flow diagram of the implementation methodology for OD flow estimation.

A complete flow diagram for the model implementation along with data pre-processing steps is shown in Figure 5. The functionality of each module is explained in the next steps.

#### 4.2.1. Microscopic Simulation

SMARTS accepts consecutive sequences of unique OSM nodes of a vehicle’s trajectory, a type of the vehicle, and a start time in a particular format. A dedicated module has been developed to perform the route generation and scheduling for the SMARTS simulation platform.

#### 4.2.2. Route Generation and Scheduling:

Each vehicle must have a complete route before being passed to the simulation platform. PTV GTFS data have been used to construct tram and bus routes. A route generator and scheduler script uses GTFS data to convert it as a sequence of consecutive OSM IDs with time-stamps for public transportation. A separate script has been used for private vehicles to construct routes with start times from estimated arrival rates. Then the public and private transport routes are integrated together. Thus, the route generating and scheduling module converts all the vehicles’ origins and destination information to scheduled and simulation-compatible routes.

#### 4.2.3. Road Network Graph:

**Figure 6:**
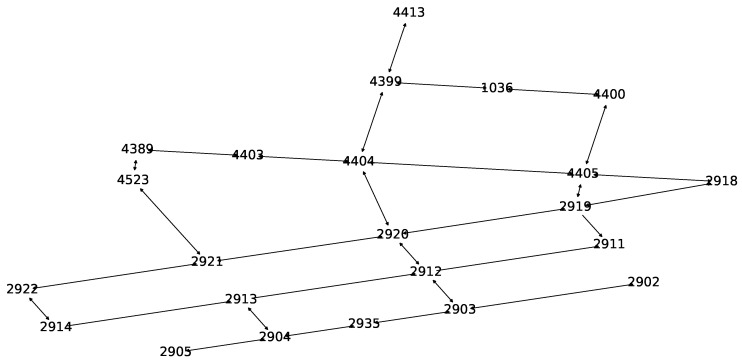
SCATSgraph.

**Figure 7:**
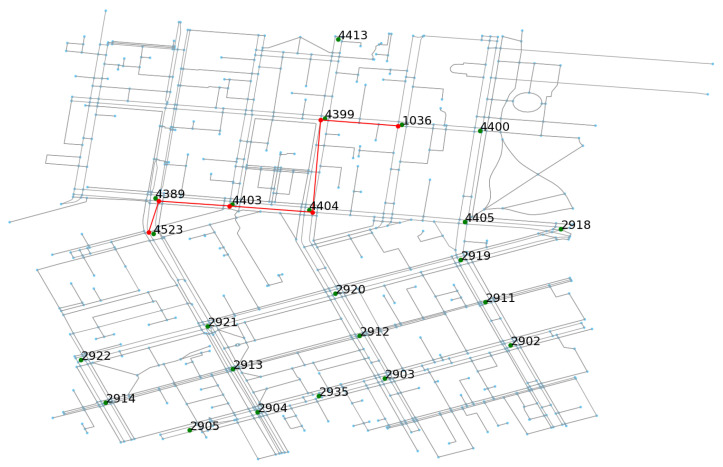
SCATS based path on top of the OSM-based bipartite graph.

A directed graph has been generated for the study area based on unique road intersection IDs as nodes and possible lanes in between as links. Weights of the edges are assigned as Euclidean distances. Figure 6 shows the SCATS graph constructed for estimating traffic generation rates of vehicles at the origins of each pair of the road intersections. The SMARTS simulation platform, however, requires a road network constructed as a directed graph where each node has a unique ID imported from the OSM database. Since each road intersection and link is typically associated with multiple OSM IDs, edge-contraction of the OSM graph has been done to reduce computational complexity in the network. A bipartite road network graph is created to assign at least one OSM ID from each lane connected to a road intersection that has a unique SCATS ID. The role of the bipartite graph is transforming calculations performed in the SCATS graph to the OSM graph for generating vehicles’ routes for the simulation platform. As an example, Figure 7 shows a SCATS ID based OD path, constructed on top of the bipartite road network graph (OSM graph) with the red lines. The green dots are the road intersections (SCATS ID).

#### 4.2.4. Traffic Generation Rate at Origins:

Transport network tomography estimates the rate of vehicles generated at the origins (λ) among all OD pairs in the study area for the specified time of the day, as described in Section 3.2.

## 5. Validation

The necessary condition for the correctness is that all the estimated OD flows must satisfy Equation (8). However, this condition is not a sufficient condition for validating OD flow because OD flow estimation is an inverse problem that has multiple solutions. In an OD flow estimation, we seek to use the most probable solution among all possible solutions. Hence, the estimated OD flow ($X^{k}$) will be always consistent with link counts ($Y^{k}$) by construction of Equation (8). This means, the OD flows cannot be validated using the same link count data used to estimate the OD flows. Thus, existing literature adopts two types of validation techniques:

- Estimated OD flow has been compared with a sample of known OD flow for a part of the city [1,9,19,20,24]. However, these observed samples only contain measured OD-flow information for a category of vehicles excluding all other vehicles on the road [1].
- Another approach for validating OD flow is done by simulating synthetic data in a controlled environment [10,12,23,26].

First we validated our model using simulated data generated from real-world random traffic, following other literature. Then, in a new approach, we validated our model using real-world data collected from two different sources. We will now discuss in detail these two necessary validation techniques in our proposed methodology.

### 5.1. Validation with Simulated Data

**Figure 8:**
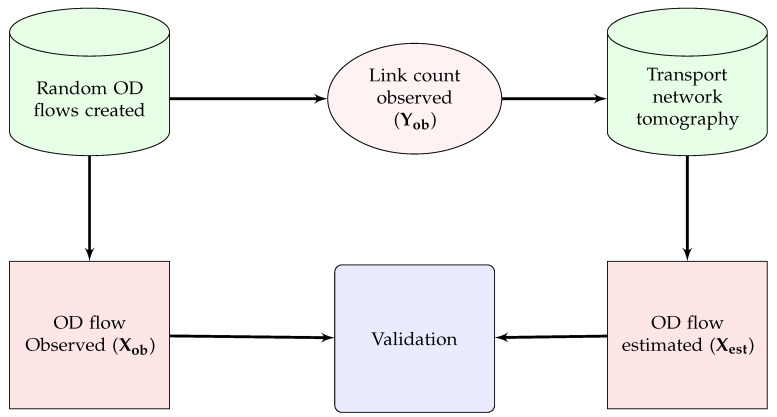
Validation technique for OD flow using real-world random traffic.

**Figure 9:**
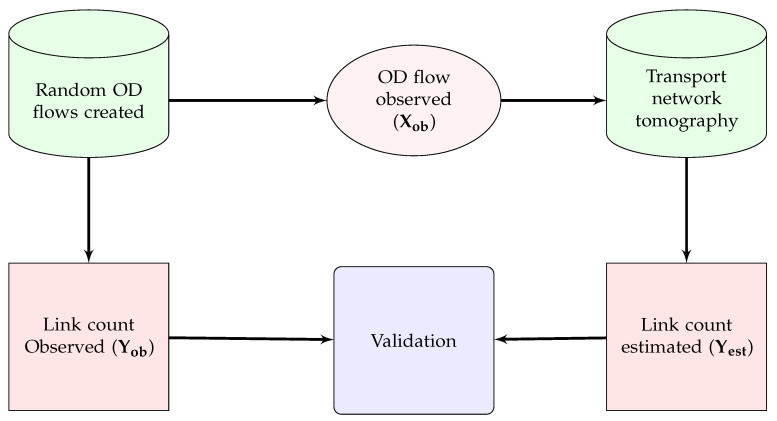
Validation technique for link count using real-world random traffic.

We have provided two validations using simulated data generated from a random traffic in the real world. We have used SMARTS to generate random OD flows in the same road network we have used to implement our model. Then we have extracted two data sets from the random OD flows: (i) observed OD flows, i.e., the number of vehicles travelling among different OD pairs, $X_{\mathrm{ob}}$, and (ii) observed link counts, i.e., the number of vehicles crossing the location of count sensors to calculate observed traffic at each link, $Y_{\mathrm{ob}}$. Then, we estimated $X_{\mathrm{est}}$ for validating with $X_{\mathrm{ob}}$ as shown in Figure 8. Our model also estimates link counts, $Y_{\mathrm{est}}$, using the inverse of Equation (8) using $X_{\mathrm{ob}}$. Thus, we get another validation of $Y_{\mathrm{est}}$ with $Y_{\mathrm{ob}}$. A flow diagram of this validation is shown in Figure 9.

### 5.2. Validation with Real World Data

The complete validation of estimated OD flows can be only done when the OD flow is fully observable, which is unrealistic for any environment [1,7,19,20,24]. Earlier literature suggested that movement in a city may be validated using travel times from trajectory data [33,37]. We have used this proposition as our second necessary validation technique. By developing this technique, we have compared estimated travel times among all OD pairs from our proposed model with observed travel times from the real world. Accordingly, we have used two sets of real-world data for validating estimated OD flow: (i) Sygic trajectory data (collected by a GPS navigation app) [42,43], and (ii) Uber movement data [25]. We have validated our model using available data for each weekday of the first quarter of the years 2016 and 2019.

#### 5.2.1. Sygic Data

We have access to Sygic trajectory data [42] of the weekdays of the first quarter (January to March) of the year 2016. Each Sygic trajectory dataset is an array of tuples where each tuple contains a geographic location (latitude and longitude), time-stamp, unique device ID, and trajectory ID of the travelled device. First the travel time along a map-matched route (within the selected area) was calculated from the observed Sygic trajectory data. Then estimated travel times for the same trajectories for same hour of a day were calculated using our proposed model. Thus, a comparison was made between the historically observed travel times with the estimated travel times for validation purposes. We had access to total 530 trajectories from each weekday of Quarter 1 of the year 2016 for validation.

#### 5.2.2. Uber Movement Data

**Figure 10:**
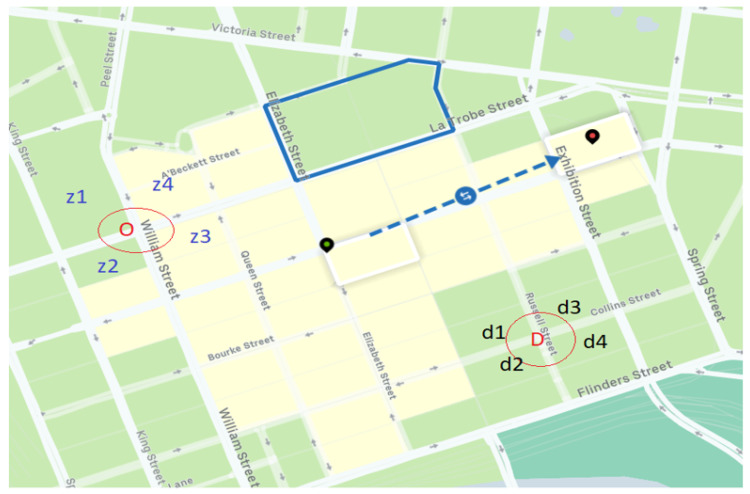
A screenshot from Uber movement data with an overlaid illustration of zonal travel times [25].

The Uber movement dataset [25] consists of means and standard deviations of travel times among OD zones (or movement id) of selected cities in the world for each hour of a day. In Figure 10, a screenshot of the base map is borrowed from the Uber movement website [25] and origins and destinations are annotated on top of the base map for visualisation purposes. In the Uber movement dataset, a geographic place of interest has been divided into convex polygons. These convex polygons are travel zones with unique identification numbers, as shown in Figure 10. These polygons are kept in the database either as origins or as destinations for each hour of a day along with the means and standard deviations of travel times. We define $\mu_{O,D}$ and $\sigma_{O,D}$ as the mean and standard deviation of observed travel times respectively from origin *O* to destination *D*. For example, in Figure 10 the green and red location markers were selected as origin and destination zones to extract travel times. However, for the current study our estimated travel times were in terms of OD node pairs. Hence, we have converted the observed zone based travel times to nodal travel times, as illustrated by red circles in Figure 10. For each OD node pair, we have aggregated the mean and standard deviation of all neighbouring OD pair zones. As an example, in Figure 10, for OD pair (O,D):

(31) $$\begin{array}{c} \mathrm{mean}\;\mathrm{travel}\;\mathrm{time}=E[\mu_{z1,d1},\mu_{z1,d2},\cdots ,\mu_{z4,d3},\mu_{z4,d4}]\\ \mathrm{standard}\;\mathrm{deviation}\;\mathrm{of}\;\mathrm{travel}\;\mathrm{time}=E[\sigma_{z1,d1},\sigma_{z1,d2},\cdots ,\sigma_{z4,d3},\sigma_{z4,d4}] \end{array}$$

We have used Equation (31) to calculate travel times among all possible OD pairs in the study area. Then, we have used every weekday of Quarter 1 of 2016 and 2019 for our model validation.

#### 5.2.3. Error Measures

Let $t_{est}$ be the estimated travel time from the model output, and $T_{mean}$ and $T_{std}$ the mean and standard deviation of travel time extracted from real-world observations (Sygic data and Uber movement data) for a particular time interval of a day for an OD pair. $N_{True}$ = number of OD pairs where $t_{est}$ is the confidence bound ($T_{mean},T_{std}$), and *N* is the set of OD pairs available for validation. We have applied the following three error measures for the estimated travel time compared to the observed travel time (Table 1): CBPE determines the percentage of estimated outputs outside the confidence interval of real-world observations. RMSE and MAPE from the mean of the observed data determine errors between estimated travel time and mean observed travel time for an OD pair.

## 6. Results

### 6.1. Validation Using Simulated Data

**Figure 11:**
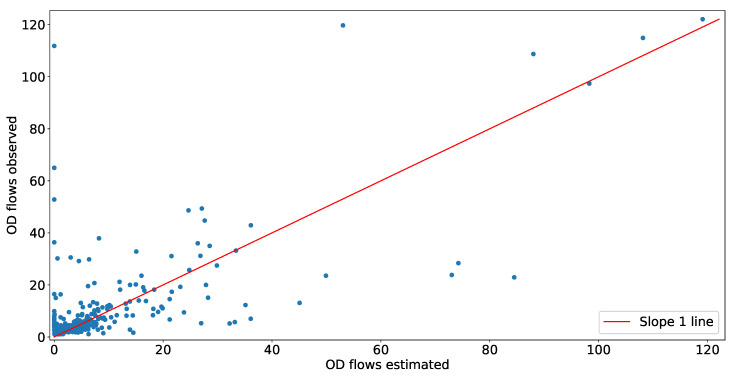
Scatter plot to compare estimated OD flow with the observed OD flow.

**Figure 12:**
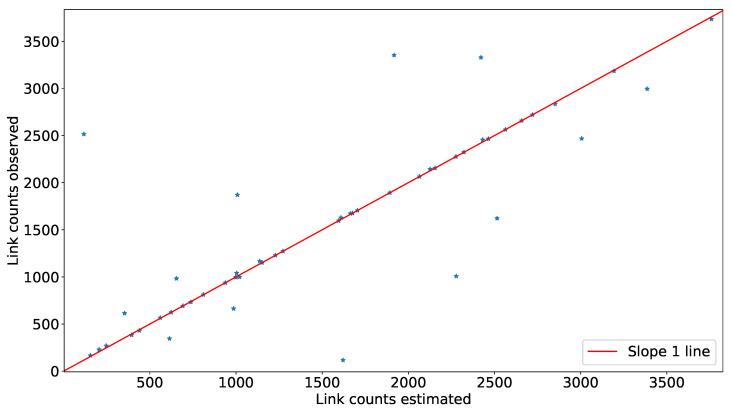
Scatter plot to compare estimated link count with the observed link count.

In this section, we validate our model using data generated from a random traffic in the same network where we have implemented our model. Our network has 484 OD pairs and 54 links. We have randomly generated OD flows for 3 h, divided into 15 min period. Figure 11 is showing a scatter plot obtained using the validation technique provided in Figure 8. In Figure 11, the y-axis represents the number of vehicles observed from the simulated data for each OD pair, and the x-axis represents the number of vehicles estimated for the same OD pair using transport network tomography. The MAPE value of the estimate is 3.11. The average observed arrival rate $E[\lambda_{ob}]$ among OD pairs is 8.67/15 min, in comparison to the average estimated arrival rate $E[\lambda_{est}]$ among the same OD pairs, which is 8.15/15 min. With the small difference, we conclude our model can estimate Λ with good accuracy. Figure 12 is showing a scatter plot as obtained using the validation technique provided in Figure 9. In Figure 12, the y-axis represents the number of vehicles observed at a particular link from the random OD flows, and the x-axis represents the number of vehicles estimated for the same link using transport network tomography. The MAPE value of the estimate is 24.18 vehicles where an average observed link count is 1567 per link.

### 6.2. Validation Using Real World Travel Time Data

As mentioned in Section 5.2, we have used two sets of historical travel time data (Uber movement and Sygic trajectory data) for our model validation. However, as these two datasets were obtained from different sources, we first cross-validated travel times between them. After that, we validated our proposed model estimated travel time with both of these observed data set separately.

#### 6.2.1. Cross Validation of Observed Data

**Figure 13:**
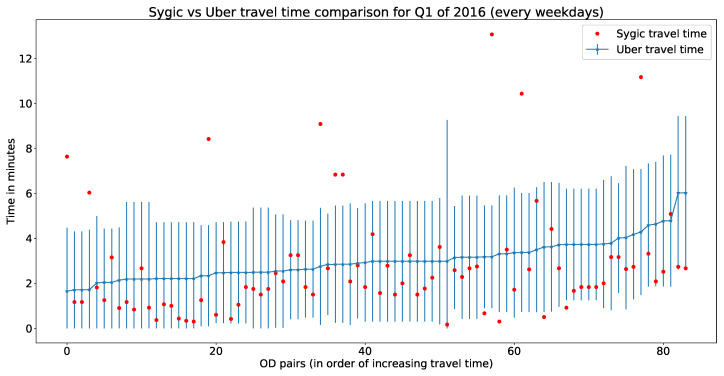
Sygic travel time data compared to Uber movement data.

To cross-validate Sygic trajectory data with Uber movement data, we used the same OD pairs and their corresponding travel times for each hour of a day (for each weekday within the first quarter of 2016). Figure 13 represents a comparative illustration of Sygic travel time with Uber movement data. The x-axis represents unique OD pairs (arranged in order of increasing Uber movement travel time), and the y-axis represents travel time (for Uber data with mean and standard deviation). In Figure 13, 88.8% of Sygic data lie within the standard deviation of the Uber movement data. Hourly errors of this comparison have been presented in Table 2.

#### 6.2.2. Estimated Travel Time Validation with Uber Movement Data

**Figure 14:**
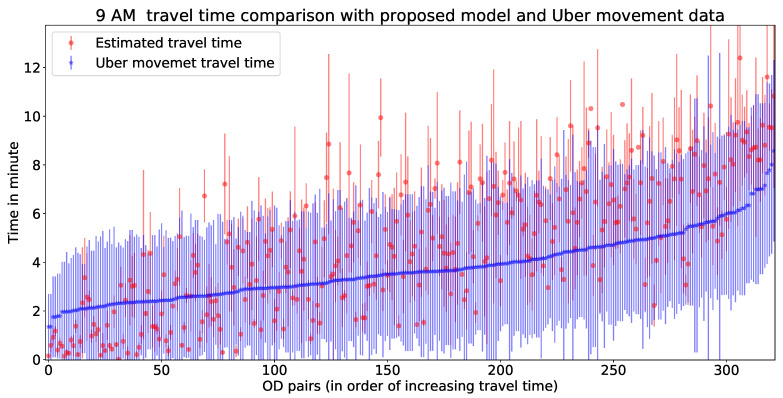
Estimated vs. Uber travel time comparison at 9 AM.

**Figure 15:**
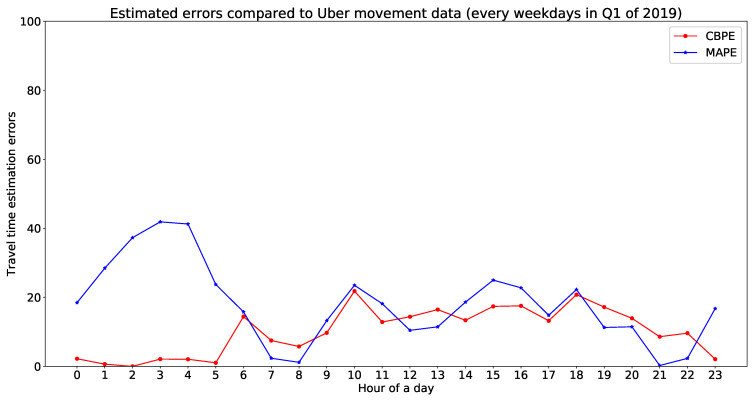
Different errors for the proposed model estimated travel time compared to Uber movement data.

In this part, we validate our model estimated travel time with Uber movement data. There are more than 300 trajectories available for hourly validation. For visualisation purpose, we have shared Figure 14 as one example to illustrate the fit of estimated travel times with Uber movement data. Figure 14 represents a comparative illustration of estimated travel time with Uber movement data. The x-axis represents unique OD pairs (arranged in order of increasing Uber movement travel time), and the y-axis represents travel time (for Uber data with mean and standard deviation). Hourly errors of this comparison have been presented in Table 2. The errors for Figure 14 can be found in the 10th row of Table 3. This implies that more than 90% of the estimated travel times lie within the standard deviation of Uber movement travel time. The rest of the hourly errors of from the validation are presented in Table 3. Figure 15 is showing a validation of estimated travel time with Uber movement data with an error plot.

#### 6.2.3. Estimated Travel Time Validation with Sygic Data

**Figure 16:**
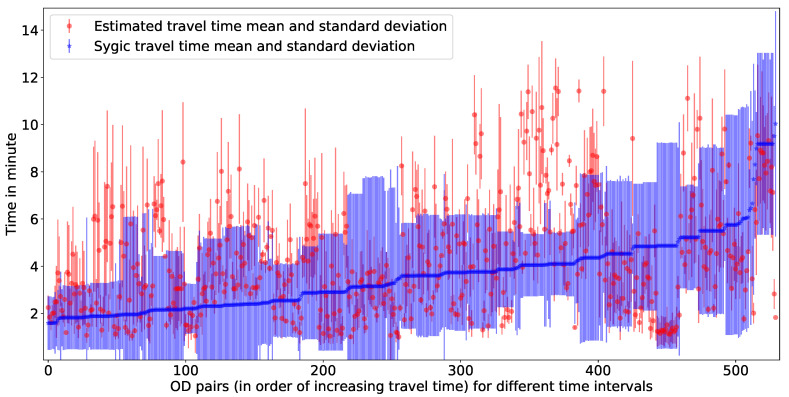
Estimated travel time validated with Sygic data (for Q1 of the year 2016).

**Figure 17:**
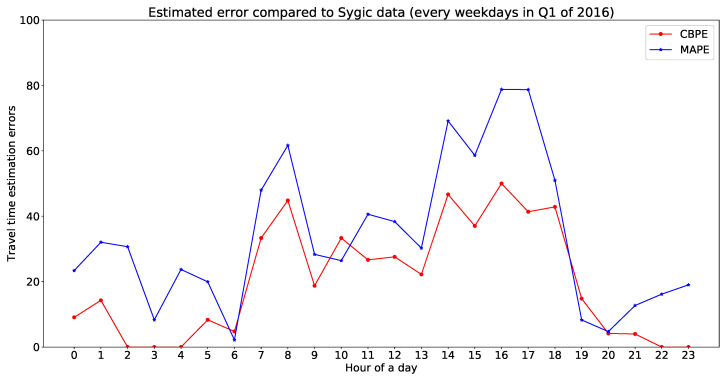
Different errors for the proposed model estimated travel time compared to Sygic data.

A total of 530 map-matched [44] trajectories have been extracted from the Sygic database (with a minimum trajectory duration of 1 min) with a sampling rate of five seconds. All of these trajectories are inside the study area shown in Figure 4. First, the travel time for each trajectory has been calculated from the observed trajectories along with their origin, destination, and OD paths. Then, the observed travel times are compared with the estimated travel time by our proposed model. Figure 16 is representing a comparative illustration of estimated travel time with Sygic data. The x-axis represents unique OD pairs (arranged in order of increasing Sygic travel time), and the y-axis represents travel time (for Sygic data with mean and standard deviation). In Figure 16, more than 75% of estimated travel times are within the standard deviation of the Sygic data. The MAPE, CBPE, and RMSE of this validation is 2.44 , 25.09, and 29.78 min respectively. Figure 17 is showing a validation of estimated travel time with Sygic data with an error plot. Hourly errors of the validation are presented in Table 4.

#### 6.2.4. Discussion

In the previous section, validation results show that a network tomography model gives an approximation of the real-world traffic flow. We have validated and explained different errors in detail. Table 5 is a comparison of mean and standard deviation of hourly errors (from Table 2, Table 3 and Table 4) of a day for all the observed and estimated travel times. In Table 5, row Sygic vs. Uber was obtained by validating two real sets of observed data. The remainder of the rows were obtained by comparing estimated output with real-world observations separately. It may be noted from Table 5 that estimated travel times agree with real-world observed travel times for the majority of the vehicles with different hourly OD flows. The results also suggest that an average of three months of link data is capable of predicting OD flows with travel times of individual vehicles.

In summary, our approach provides an estimation of OD flow using link count data only along with an estimation of the historical travel times for individual vehicles. We have also shown that traffic generation rates at the origins in a small time interval are capable of predicting historical traffic conditions in a city. The estimation technique performs well for predicting a large range of travel times in the constrained test area.

## 7. Conclusions and Future Work

In this paper, we proposed a method to estimate and validate OD flows only from link count data observed at road intersections. Our model can also estimate the travel times of individual vehicles in the road network from the the estimated OD flows. The approach is based on the network tomographic model of traffic flow in communication networks, and has been integrated with time-geographic constraints, thereby overcoming some of the limitations in all existing approaches. The model has been implemented over a part of the centre of Melbourne. The model has been validated first by generating random OD flows in the real-world network. Then, the model estimated travel times were validated against the travel times obtained from two sets of real-world data.

Validation results show that under certain choices of model parameters, urban traffic OD flow along with travel time can be estimated with reasonable accuracy at a microscopic scale. Particular choices of different parameters and assumptions (e.g., number of iterations in the EM algorithm, total number of weekdays, interval time, regularisation constant) used in the model may influence the simulated results and prevent over-fitting of the estimated data. This choice of model parameter is for those road intersections and road links that are geographically distant from each other; hence, aim to minimise the influence of the link co-variances in the estimation. In future work, the investigation of other parts and further characteristics of road networks will illuminate the limits to which the developed methodology is generic. Further investigations will also show whether other choices of model parameters might estimate the traffic flow with increased accuracy. The model as shows good accuracy in estimating travel time for different duration of one minute to 15 min. However, the model was not evaluated for trajectories with longer/shorter duration. The size of the road network used for the implementation was not extended due to missing link count data outside of this area. Recently, many other sensors were adopted to estimate passenger flow, and also to evaluate the accuracy of flow estimates. Accordingly, as a future work, our model can be extended for tracking passengers throughout their journeys on public transportation vehicles using automatic passenger counting systems [45,46].

## Figures and Tables

**Table 1:** Definitions of applied error measures.

Confidence Bound Percentage Error (CBPE)	$\frac{N_{True}}{N}*100\%$
Root Mean Squared Error (RMSE) from the Observed Mean	$\sqrt{\frac{1}{N}\sum_{1}^{N}{\left(t_{est}-T_{mean}\right)}^{2}}$
Mean Absolute Percentage Error (MAPE) from the Mbserved Mean	$\frac{100\%}{N}\sum_{1}^{N}\left|\frac{t_{est}-T_{mean}}{T_{mean}}\right|$

**Table 2:** MAPE, CBPE, and RMSE errors of Sygic data compared to Uber movement data for different hours of a day.

	MAPE	CBPE	RMSE	Hod
1	17.669511	15.517241	2.645850	1.0
2	0.202307	15.740741	2.445008	2.0
3	0.718313	14.432990	2.393097	3.0
4	22.953926	19.767442	2.329059	4.0
5	26.719827	20.535714	3.328379	5.0
6	12.443983	11.538462	2.206808	6.0
7	14.955032	13.445378	2.081354	7.0
8	27.649186	13.157895	2.963124	8.0
9	32.979581	24.299065	3.858327	9.0
10	1.480969	12.222222	3.080802	10.0
11	2.016291	19.642857	2.882764	11.0
12	1.424106	24.637681	3.278605	12.0
13	1.534412	25.000000	3.168098	13.0
14	16.658874	23.404255	2.993369	14.0
15	22.793953	12.121212	2.591443	15.0
16	9.032345	6.666667	2.953414	16.0
17	30.181544	13.043478	2.855195	17.0
18	1.401953	46.666667	3.206350	18.0
19	28.902722	5.555556	1.326432	19.0
20	10.646802	16.129032	2.375741	20.0
21	31.177547	8.000000	1.634604	21.0
22	16.119596	23.376623	2.923352	22.0
23	4.494027	15.476190	2.502182	23.0
24	4.236363	12.359551	2.608609	24.0

**Table 3:** CBPE, RMSE, and MAPE errors of estimated travel time compared to Uber movement data for different hours of a day.

	MAPE	CBPE	RMSE	Hod
0	18.456364	2.212389	1.086122	0.0
1	28.449028	0.641026	1.071584	1.0
2	37.291042	0.000000	1.142930	2.0
3	41.875131	2.105263	1.234366	3.0
4	41.253799	2.061856	1.149237	4.0
5	23.741862	1.036269	1.096324	5.0
6	15.789029	14.418605	1.864147	6.0
7	2.361422	7.490637	1.570877	7.0
8	1.162064	5.769231	2.244866	8.0
9	13.253511	9.717868	2.024412	9.0
10	23.498247	21.806854	2.083335	10.0
11	18.150688	12.852665	2.046035	11.0
12	10.440517	14.375000	2.049898	12.0
13	11.458377	16.455696	2.454868	13.0
14	18.620275	13.354037	2.210899	14.0
15	24.994183	17.378049	2.663201	15.0
16	22.769228	17.538462	2.538258	16.0
17	14.798218	13.213213	2.722560	17.0
18	22.258559	20.783133	2.602251	18.0
19	11.269477	17.177914	1.846150	19.0
20	11.470420	13.931889	1.753256	20.0
21	0.246007	8.598726	1.601560	21.0
22	2.320536	9.615385	1.837532	22.0
23	16.737412	2.090592	1.323046	23.0

**Table 4:** CBE, RMSE, and MAPE errors of estimated travel time compared to Sygic data for different hours of a day.

	CBPE	MAPE	RMSE	Hod
0	9.090909	23.380651	1.568953	1.0
1	14.285714	32.053831	2.062144	2.0
2	0.000000	30.691456	1.983764	3.0
3	0.000000	8.316054	0.779859	4.0
4	0.000000	23.730673	1.332108	5.0
5	8.333333	19.952337	1.948909	6.0
6	4.761905	2.174713	1.738910	7.0
7	33.333333	47.974776	2.624935	8.0
8	44.827586	61.672828	3.215480	9.0
9	18.750000	28.348935	1.984638	10.0
10	33.333333	26.419977	2.746909	11.0
11	26.666667	40.617233	2.151389	12.0
12	27.586207	38.360967	2.357824	13.0
13	22.222222	30.253156	2.071730	14.0
14	46.666667	69.130894	3.357464	15.0
15	37.037037	58.616123	2.701654	16.0
16	50.000000	78.787198	3.434253	17.0
17	41.379310	78.718194	3.540826	18.0
18	42.857143	50.967232	2.666515	19.0
19	14.814815	8.287783	1.671548	20.0
20	4.166667	4.728311	1.502707	21.0
21	4.000000	12.711000	1.491700	22.0
22	0.000000	16.183482	1.363504	23.0
23	0.000000	19.018031	1.418765	24.0

**Table 5:** Means and standard deviations of hourly errors of a day.

	Error	MAPE		CBPE		RMSE	
Data							
		Mean	Standard deviation	Mean	Standard deviation	Mean	Standard deviation
Sygic vs Uber		14.1	11.48	17.19	8.43	2.6	0.55
Estimated vs Uber		18.02	11.54	10.2	6.87	1.84	0.54
Estimated vs Syic		33	22.7	20.1	17.4	2.1	0.7

## Abbreviations

*G*	Road network graph
*n*	Number of nodes in *G*
*J*	Maximum possible OD pair in *G*
*I*	number of links/edges in *G*
*T*	A constant time interval of a day
$X_{j}^{k}$	Number of cars travelling between OD pair j at $k^{\mathrm{th}}$ time interval
$Y_{j}^{k}$	Number of cars observed at a link i at $k^{\mathrm{th}}$ time interval
$\lambda_{j}$	Rate of cars originating between OD pair *j*
$a_{i}^{j}$	Probability of a car observed at a link *i* belongs to OD pair *j* without the knowledge of previous link
$P_{i}^{j}$	Probability of a car observed at a link *i* belongs to OD pair *j* with the knowledge of previous link
E[X]	First order moment of a random variable *X*
*A*	Initial routing matrix with memory less Marokvian assumption
*P*	Route choice matrix with finite memory Marokvian assumption
$Q^{\left(m\right)}$	$m^{\mathrm{th}}$ order state transition matrix
S,B	Co-variance matrices
